# Molecular Subtype Classification of Postmenopausal Osteoporosis and Immune Infiltration Microenvironment Based on Bioinformatics Analysis of Osteoclast-Regulatory Genes

**DOI:** 10.3390/biomedicines11102701

**Published:** 2023-10-04

**Authors:** Yining Gong, Dingjun Hao, Yong Zhang, Yongyong Tu, Baorong He, Liang Yan

**Affiliations:** 1 Department of Spine Surgery, Honghui Hospital, Xi’an Jiaotong University, Xi’an 710054, China; gong_yn@bjmu.edu.cn (Y.G.); 1310301542@bjmu.edu.cn (D.H.); zechaoqu@yau.edu.cn (Y.Z.); 1510301104@pku.edu.cn (Y.T.); 1310301541@bjmu.edu.cn (B.H.); 2 Institute of Orthopedic Surgery, Honghui Hospital, Xi’an Jiaotong University, Xi’an 710054, China

**Keywords:** osteoporosis, molecular subtype, risk stratification, immune infiltration microenvironment, bioinformatics, osteoclast

## Abstract

Osteoporosis is common in postmenopausal women but is often asymptomatic until a fracture occurs, highlighting the importance of early screening and preventive interventions. This study aimed to develop molecular subtype risk stratification of postmenopausal osteoporosis and analyze the immune infiltration microenvironment. Microarray data for osteoporosis were downloaded and analyzed. Logistic and least absolute shrinkage and selection operator (LASSO) regression analyses were used to construct the molecular risk model. Circulating blood samples were collected from 10 enrolled participants to validate the key differentially expressed genes, and consistent clustering based on the expression profiles of candidate genes was performed to obtain molecular subtypes. Three key genes, *CTNNB1*, *MITF*, and *TNFSF11*, were obtained as variables and used to construct the risk model. External experimental validation showed substantial differences in the three key genes between patients with osteoporosis and the controls (*p* < 0.05). Three subtypes were obtained based on dimensionality reduction clustering results. Cluster 3 had significantly more patients with low bone mineral density (BMD), whereas Cluster 2 had significantly more patients with high BMD (*p* < 0.05). This study introduced a novel molecular risk model and subtype classification system, which is an evidence-based screening strategy that will guide the active prevention, early diagnosis, and treatment of osteoporosis in high-risk postmenopausal women.

## 1. Introduction

Osteoporosis is a systemic, aging-related, skeletal disease characterized by low bone mass and microstructural destruction [1]. In clinical practice, osteoporosis is diagnosed as a bone mineral density (BMD) T-score of −2.5 or less [2]. Osteoporosis leads to increased bone fragility and greater susceptibility to fractures, thereby adversely affecting orthopedic surgery because of the high incidence of implant failure [3]. Epidemiological studies have shown that the prevalence of osteoporosis in postmenopausal women is as high as 32.1% [4]. In addition, owing to its age-related characteristics and the aging of the global population, the number of patients with osteoporosis is expected to increase rapidly, resulting in higher complication rates, mortality, and medical burden [5,6].

Osteoporosis is common in postmenopausal women but is often asymptomatic until fractures occur. From the perspective of disease characteristics and cost-effectiveness, early screening and preventive interventions are required [7,8]. There are many options for treating osteoporosis, including anti-resorptive and anabolic medications, as well as some natural compounds [9]. However, knowledge of screening and prevention has not developed at the same rate as diagnosis and treatment advances in recent years. Dual-energy X-ray absorptiometry (DXA) of the hip and lumbar spine is the most widely used tool for the diagnosis of osteoporosis, and is the recommended test of BMD screening for all women aged 65 years or older by the US Preventive Services Task Force and other professional societies [10,11]. However, in practice, it is difficult to screen the entire at-risk population using DXA without triage tests [10]. Several epidemiological studies have developed clinical decision-making tools for osteoporosis risk assessment to screen postmenopausal women for BMD measurements [12,13,14]. However, there is still room for these decision-making tools based solely on clinical information when it comes to their sensitivity and specificity [15].

Both genetic and environmental factors contribute to the development of osteoporosis, but compared with numerous previously reported clinical tools, risk assessment models of osteoporosis based on genetic information are rare [16]. Since the clinical importance of the genetic determination of osteoporosis is incontestable, a study of molecular risk models based on regulation genes can be a timely and interesting contribution. Osteoclasts play an important role in maintaining the balance of bone metabolism and have been extensively studied in osteoporosis [17]. Previous studies have reported a few genetic markers associated with osteoporosis [18,19]. Comparisons between healthy and diseased individuals at the transcript level facilitate the identification of differentially expressed genes (DEGs), which may indicate disease causes or consequences and other factors correlated with the disease under scrutiny [20]. In the present study, we aimed to further stratify the risk of osteoporosis in postmenopausal women based on DEGs. Although the immune infiltration microenvironment is closely related to the development of osteoporosis, little is known about its relationship with patients at different risk levels [21]. Through this study, we also intended to speculate on the differences in the immune infiltration microenvironment in patients with different molecular subtypes.

To address these needs, in this study, we constructed a novel molecular risk model and molecular subtypes in postmenopausal women by identifying DEGs in osteoclasts. Such evidence-based screening strategies will guide the active prevention, early diagnosis, and treatment of osteoporosis for high-risk postmenopausal women.

## 2. Materials and Methods

### 2.1. Public Dataset Source and Processing

GSE56815, a microarray of osteoporosis data of circulating monocytes, was downloaded from the Gene Expression Omnibus (GEO) using the R package “GEOquery”, comprising a total of 80 samples from 40 female patients with high BMD and 40 with low BMD. The data were pre-processed as follows: the probes corresponding to genes were retained and the no-load probes were removed; multiple probes corresponding to the same gene were then selected as average gene expression levels. The functions and corresponding gene sets related to osteoclast regulation were obtained from the Molecular Signatures Database (MSigDB). The gene expression profile data for these osteoclast-regulatory genes were selected from the GSE56815 dataset for further differential analysis.

### 2.2. Participants

This study was approved by the institutional review board of Honghui Hospital (no. 202206029) and was performed according to the Helsinki Declaration of 1975, as revised in 2000. Written consent was obtained from all participants included in the study. Ten participants were enrolled in this study, with five each in the osteoporosis and control groups. Circulating blood was collected from all participants. These participants underwent spine surgery for degenerative lumbar disease in the hospital and were evaluated by a senior osteoporosis specialist. Details of the participants are provided in the Results section. All patients completed the Oswestry Disability Index and Karnofsky Performance Status scales at the time of enrollment to minimize the influence of the external factors of daily life on BMD. All patients were evaluated by a senior osteoporosis specialist for primary osteoporosis.

### 2.3. Identification of DEGs and Construction of the Molecular Risk Model

The “limma” package in R software (version 4.1.1, R Foundation for Statistical Computing, Vienna, Austria) was used to analyze the DEGs between high- and low-BMD samples in the GSE56815 dataset. Heatmaps and boxplots of DEGs were produced using R software (version 4.1.1) and the packages “pheatmap” and “ggplot”. Univariate and multivariate logistic regression analyses were conducted to identify candidate genes related to BMD among the DEGs. The LASSO regression model reduces the dimensionality of the data to reduce noise or redundant genes. Candidate genes were subjected to LASSO regression analysis using the R package “glmnet”. We used family = “binomial” to fit the model, and the most effective candidate genes and the optimal value of the penalization λ were determined via 10-fold cross-validation. The risk score of each patient with osteoporosis was calculated using the corresponding regression coefficients and the expression levels of the candidate genes. The calculation formula is as follows:

$$score=\sum_{i=1}^{n}\left({coef}_{i}\times {expr}_{i}\right),$$

where ${coef}_{i}$ is the LASSO regression coefficient and $\;{expr}_{i}$ is the expression value of the gene. We divided the patients with osteoporosis into high- and low-risk groups based on the median value. The area under the receiver operating characteristic curve was calculated using the R package “pROC” to evaluate the prediction performance.

### 2.4. External Experimental Validation

Peripheral blood mononuclear cells (PBMCs) were isolated from the blood sample of each patient. Total RNA from PBMCs was extracted using TRIzol reagent (Ambion; Thermo Fisher Scientific, Waltham, MA, USA) following standard procedures. An optical density ratio at 260 nm/208 nm of 1.8–2.0 satisfied the experimental requirements. RNA (2 µg) from each sample was reverse-transcribed into cDNA using 5× HiScript II Select qRT SuperMix II (VAZYME, Nanjing, China). qPCR was performed with SYBR Green Master Mix (VAZYME) using an ABI QuantStudio 6 Real-Time System (Applied Biosystems, Waltham, MA, USA), according to the standard protocols, programmed to perform 42 cycles in total. Specific primers for mRNAs were synthesized by Tsingke Biotechnology (Beijing, China). Relative mRNA transcript levels were normalized to those of β-actin. The primer sequences are as follows: *CTNNB1* forward CCAAGTGGGTGGTATAGAGG, reverse AGTCCATAGTGAAGGCGAAC (156 bp); *MITF* forward CCAAAGAGAGGCAGAAAAAGGA, reverse CGTGGATGGAATAAGGGAAAGTC (311 bp); *TNFSF11* forward ATCTGGTTCCCATAAAGTGAG, reverse CGAAAGCAAATGTTGGCATA (141 bp); and β-actin forward CCCTGGAGAAGAGCTACGAG, reverse CGTACAGGTCTTTGCGGATG (180 bp). The expression level of each mRNA was calculated using the 2^−△△Ct^ method. 

### 2.5. Abundance of Infiltrating Immune Cells

The CIBERSORT algorithm uses the deconvolution method to extract features from RNA-sequencing data and inversely calculates the proportions of various cellular components in bulk-seq. The R script “CIBERSORT” was used to detect the abundance of 22 immune cells in all samples.

### 2.6. Molecular Subtypes of Osteoporosis Samples

Consensus clustering is an algorithm that can be used to identify the members of clusters and their numbers in a dataset. We used the R package “ConsensusClusterPlus” to perform consistent clustering of samples based on the expression profiles of candidate genes to obtain three molecular subtypes.

### 2.7. Functional Enrichment Analysis and Gene Set Variation Analysis

The R package “clusterProfiler” was used to perform functional enrichment analysis for DEGs of each subtype, and the significantly enriched pathways and functions were screened using a threshold of *p* < 0.05. We downloaded the Kyoto Encyclopedia of Genes and Genomes (KEGG) pathway gene set from MSigDB and used the R package “GSVA” to calculate the pathway enrichment scores for each sample. Gene set variation analysis is a non-parametric, unsupervised method for estimating enriched variation in gene sets from samples of expression datasets. The gene set file “c2. cp. kegg. v7.5.1. symbols”, containing 184 key genes, was downloaded from MSigDB.

### 2.8. PPI Network Construction and Topology Feature Analysis

We used the STRING database to map the PPI networks of 26 DEGs on human protein interaction networks in the database, and then, reconstructed the network using Cytoscape (version 3.9.1). The MCODE plugin was used to detect important co-regulation modules for sub-network analysis. The cytoHubba plugin was used to compute hub nodes.

### 2.9. Quantification and Statistical Analyses

All statistical analyses were performed using R, version 4.1.1 (R Foundation for Statistical Computing, Vienna, Austria). The Wilcoxon test was used to calculate the statistical significance of two groups of variables, and the Kruskal-Wallis test was used to calculate the statistical significance of multiple groups of variables. Spearman’s correlation coefficient was used to analyze the correlation between two groups of variables. Differences in clinical characteristics between the molecular subtypes were determined using the Chi-square test.

## 3. Results

### 3.1. Functions of Osteoclast-Regulatory Genes

**Figure 1:**
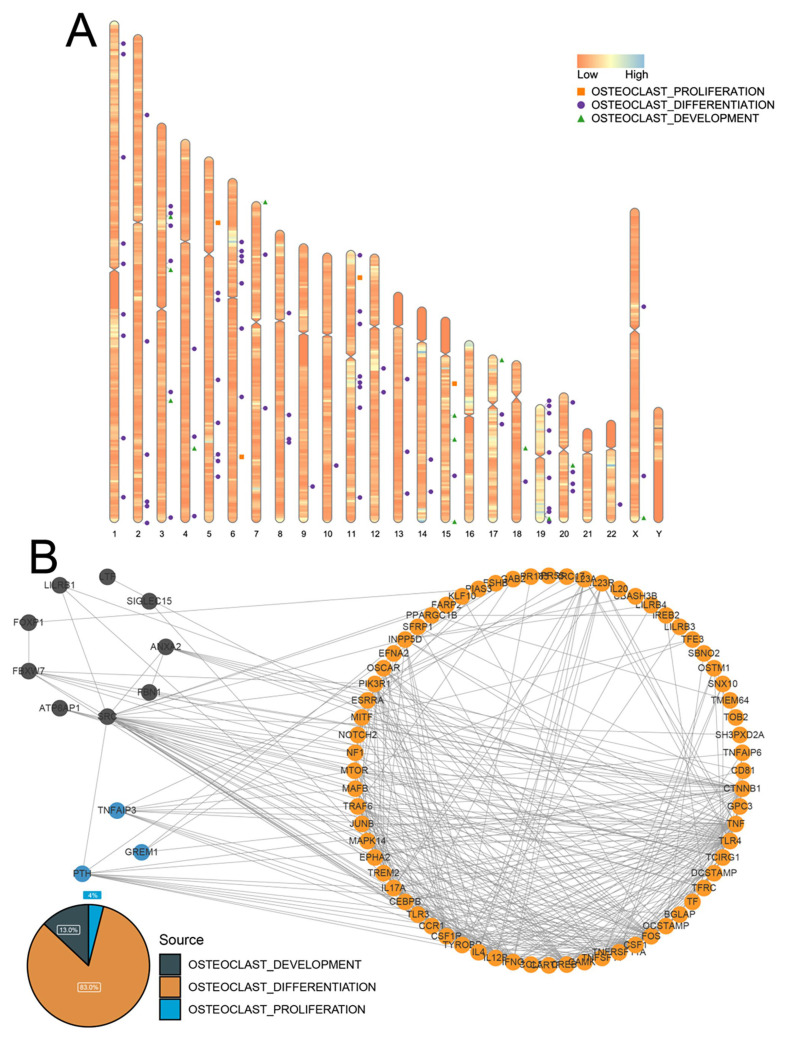
Characterization of osteoclast-regulatory genes. (**A**) The locations of genes related to osteoclast regulation on chromosomes. (**B**) Protein–protein interaction network of three types of genes related to the regulation of osteoclasts and the proportion of each type of gene: osteoclast development-related genes (black), osteoclast differentiation-related genes (orange), and osteoclast proliferation-related genes (blue).

Functions related to osteoclast regulation were downloaded from MSigDB, and nine functions related to osteoclast regulation were obtained, comprising five osteoclast differentiation-related functions, three osteoclast development-related functions, and one osteoclast proliferation-related function. These functions included 100 genes related to osteoclast regulation, comprising 83, 13, and 4 genes regulating osteoclast differentiation, development, and proliferation, respectively. Figure 1A illustrates the location of osteoclast-regulatory genes on the chromosome. We mapped the osteoclast-regulatory genes in the STRING database to obtain the interaction network of these genes. The majority of the genes were related to osteoclast differentiation, whereas the osteoclast proliferation-related genes were in the minority. Moreover, there was close interaction between the three gene groups. There was an interaction between proliferation-related genes and a large number of differentiation-related genes. In particular, this analysis suggested that development-related SRC genes may play an important role in osteoclast differentiation (Figure 1B).

### 3.2. Expression Levels and Correlation of Osteoclast-Regulatory Genes

**Figure 2:**
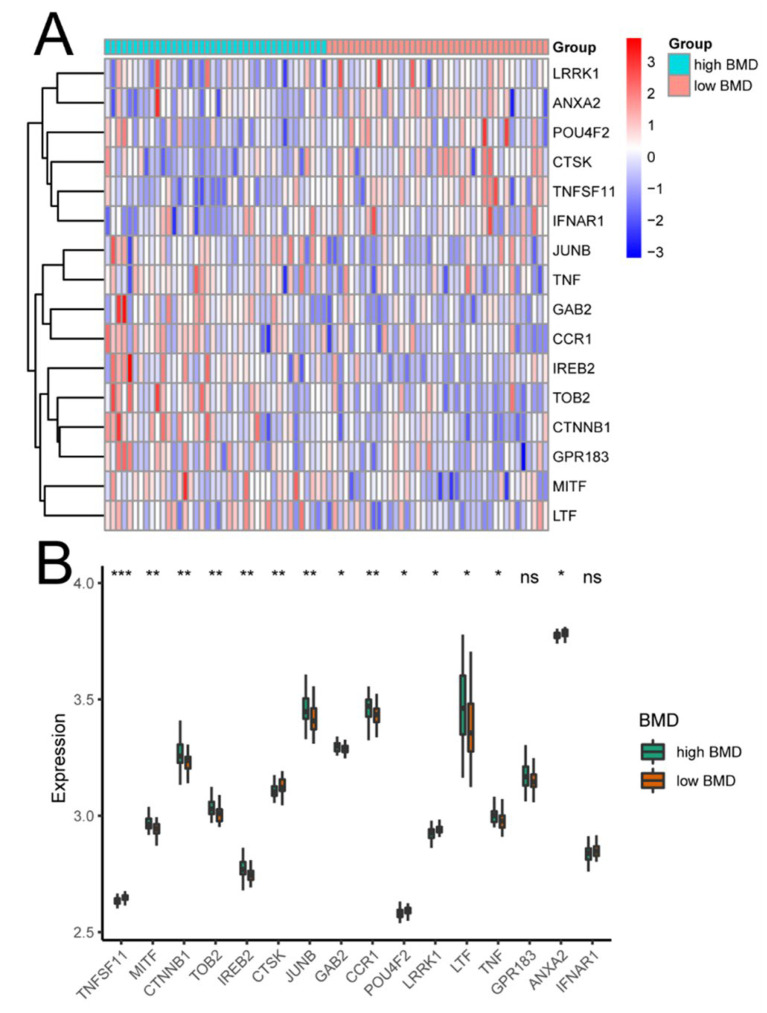
Expression levels of osteoclast-regulatory genes according to bone mineral density (BMD). (**A**) Expression heatmap of differentially expressed osteoclast regulation-related genes in patients with high (light blue) and low (peach) BMD. (**B**) Boxplots comparing the expression distribution of differentially expressed osteoclast-regulatory genes between patients with high (green) and low (orange) BMD. The Wilcoxon test was used to determine the significance of differential expression. ns: no statistically significant difference; * *p* < 0.05; ** *p* < 0.01; *** *p* < 0.001.

The gene expression profile data of 96 osteoclast-regulatory genes were selected from the GEO GSE56815 dataset based on a microarray of standardized osteoporosis data of circulating monocytes, comprising a total of 80 samples from 40 female patients with high BMD and 40 with low BMD. Sixteen DEGs were found between the high- and low-BMD groups, among which six genes (*LRRK1*, *ANXA2*, *POU4F2*, *CTSK*, *TNFSF11*, and *IFNAR1*) were substantially overexpressed in the low-BMD samples and 10 genes (*JUNB*, *TNF*, *GAB2*, *CCR1*, *IREB2*, *TOB2*, *CTNNB*, *GPR183*, *MITF*, and *LTF*) were substantially overexpressed in the high-BMD samples (*p* < 0.05) (Figure 2).

**Figure 3:**
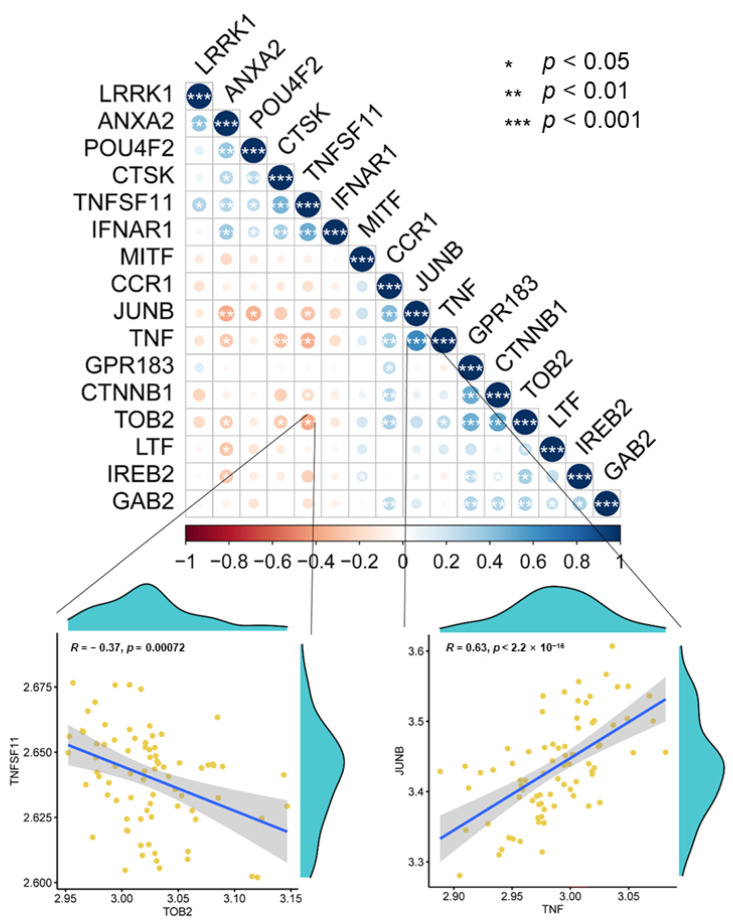
Correlation of osteoclast-regulatory gene expression. Dot plot showing the correlations between the expression levels of osteoclast regulation-related genes, analyzed via Spearman’s correlation coefficient. The correlations between *TOB2* and *TNFSF11* (R = −0.37, *p* = 0.00072) and between *TNF* and *JUNB* (R = 0.63, *p* < 2.2 × 10^−16^) are shown in detail.

Spearman’s correlation coefficient was used to analyze the correlation between the 16 differentially expressed osteoclast-regulatory genes. *TOB2* expression negatively correlated with *TNFSF11* expression (R = −0.37, *p* < 0.01), whereas *TNF* expression positively correlated with *JUNB* expression (R = 0.63; *p* < 0.001) (Figure 3). These four genes are associated with osteoclast differentiation. *TOB2* belongs to the antiproliferative protein family and is involved in the regulation of cell cycle progression. *TNF* is involved in the regulation of a wide range of biological processes, including cell proliferation, differentiation, apoptosis, and lipid metabolism. *JUNB* is involved in the positive regulation of transcription by RNA polymerase II. *TNFSF11* is a key factor in osteoclast differentiation and activation.

### 3.3. Molecular Risk Model for Osteoporosis

**Figure 4:**
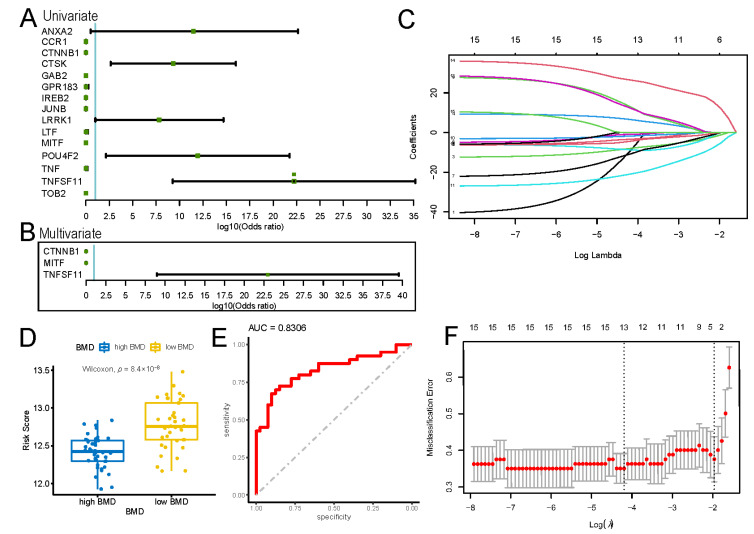
Construction of a molecular risk model for osteoporosis based on osteoclast-regulatory genes. (**A**) Univariate logistic regression results of differentially expressed osteoclast regulation-related genes. (**B**) Multivariate logistic regression results of *CTNNB1*, *MITF*, and *TNFSF11*. (**C**) Coefficient change of each feature according to the lambda change in the LASSO regression model. (**D**) Distribution of risk scores between high (blue)- and low (yellow)-bone-mass-density (BMD) groups. The Wilcoxon test was used to determine the significance of the difference in risk scores. (**E**) Receiver operating characteristic curve to evaluate risk model performance according to the area under the curve (AUC) value. (**F**) Confidence interval of the target parameter after cross-validation.

Univariate logistic regression was performed using BMD as the dependent variable and the expression level of each differentially expressed osteoclast-regulatory gene as the independent variables. Fifteen genes were found to be statistically significant, among which *CCR1*, *CTNNB1*, *GAB2*, *GPR183*, *IREB2*, *JUNB*, *LTF*, *MITF*, *TNF*, and *TOB2* emerged as protective factors, whereas *CTSK*, *ANXA2*, *LRRK1*, *POU4F2*, and *TNFSF11* emerged as risk factors (*p* < 0.05) (Figure 4A). 

Least absolute shrinking and selection operator (LASSO) regression analysis was performed on these genes to determine the coefficient change of each feature according to the lambda (λ) change; the λ and log (λ) values were set to 0.14 and −1.97, respectively (Figure 4C,F). Three of the fifteen genes, *CTNNB1*, *MITF*, and *TNFSF11*, were selected in LASSO regression to construct the risk model (risk score = Exp (*CTNNB1*) × (−1.167227) + Exp (*MITF*) × (−5.192496) + Exp (*TNFSF11*) × (12.019122)). The key roles of these three genes in osteoporosis were verified using multivariate logistic regression, with *CTNNB1* (OR = 3.501 × 10^−7^, *p* = 0.016) and *MITF* (OR = 2.475 × 10^−14^, *p* = 0.002) as protective factors and *TNFSF11* (OR = 9.245 × 10^22^, *p* = 0.003) as a risk factor (Figure 4B). These results indicated that *CTNNB1*, *MITF*, and *TNFSF11* are of great significance in the occurrence and development of osteoporosis. Indeed, patients with low BMD had significantly higher risk scores from the model and patients with high BMD had lower risk scores (*p* < 0.001) (Figure 4D). Finally, the receiver operating characteristic curve of all samples demonstrated the excellent diagnostic performance of our risk model (area under the curve = 0.8306; Figure 4E).

### 3.4. External Experimental Validation of the Key Genes in the Osteoporosis Risk Model

**Figure 5:**
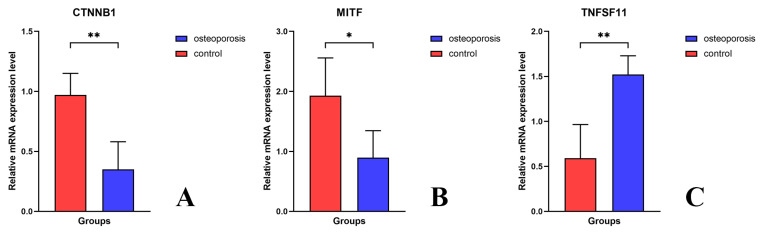
Quantitative real-time polymerase chain reaction verification of key differentially expressed genes. Compared with that in the control groups (red), the relative expression of *CTNNB1* (**A**) and *MITF* (**B**) was significantly downregulated, whereas that of *TNFSF11* (**C**) was significantly upregulated in the osteoporosis groups (blue). * *p* < 0.05; ** *p* < 0.01.

Ten patients were enrolled in this study for experimental validation, including five with and five without osteoporosis (see Table 1 for the inclusion and exclusion criteria). The average age at enrollment was 62.2 ± 7.7 years, and the average time after menopause was 15.8 ± 6.3 years. The average BMD T-values in the osteoporosis group were −3.1 ± 0.4 (spine) and −3.4 ± 1.0 (hip), and those in the control group were −1.3 ± 0.8 (spine) and −1.1 ± 0.8 (hip). There was no significant difference between the control and osteoporosis groups in terms of body mass index (26.7 ± 5.3 vs. 23.6 ± 3.5; *p* = 0.309), serum calcium level (2.3 ± 0.1 vs. 2.3 ± 0.0; *p* = 0.633), serum phosphate level (1.3 ± 0.1 vs. 1.2 ± 0.2; *p* = 0.369), Oswestry Disability Index (0.3 ± 0.2 vs. 0.5 ± 0.1; *p* = 0.087), and Karnofsky Performance Status (82.0 ± 11.0 vs. 72.0 ± 4.5; *p* = 0.114). The quantitative polymerase chain reaction (qPCR) of osteoclast regulation genes selected in the risk model in these patients showed consistent results with the analysis of public microarray data. *TNFSF11* was significantly upregulated (*p* = 0.001), whereas *CTNNB1* (*p* = 0.001) and *MITF* (*p* = 0.017) were significantly downregulated, in the osteoporosis group (Figure 5).

### 3.5. Associations of Osteoclast-Regulatory Genes with the Immune Microenvironment

**Figure 6:**
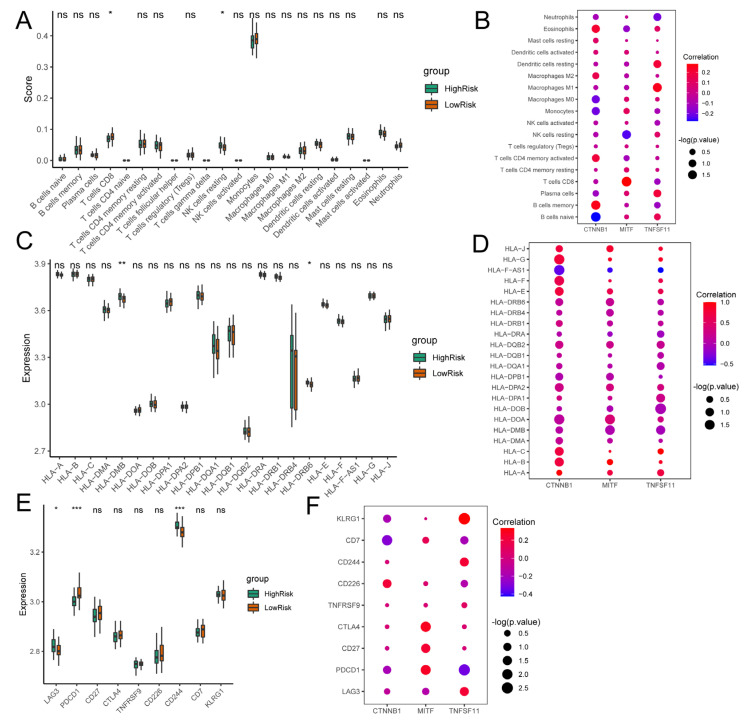
Osteoclast-regulatory genes and the immune microenvironment. (**A**) Differences in 22 types of immune cells between high- (green) and low-risk (orange) groups. (**B**) Correlations between *CTNNB1*, *MITF*, and *TNFSF11* expression and the 22 types of immune cells. (**C**) Differences in expression of HLA-related genes between high- (green) and low-risk (orange) groups. (**D**) Correlations between *CTNNB1*, *MITF*, *TNFSF11*, and HLA-related genes. (**E**) Differences in expression of immune-response-related genes between high- (green) and low-risk (orange) groups. (**F**) Correlations between *CTNNB1*, *MITF*, and *TNFSF11* expression levels and immune-response-related genes. The Wilcoxon test was used to determine the significance of immune cells and related genes between high- and low-risk groups. Spearman’s correlation coefficient was used to calculate the correlations. ns: no statistically significant difference; * *p* < 0.05; ** *p* < 0.01; *** *p* < 0.001.

After dividing the patients into high- and low-risk groups according to the median value of the risk score, we calculated the abundance of immune cells in the two groups. The low-risk group had substantially more CD8^+^ T cells and the high-risk group had substantially more natural killer (NK) cells (Figure 6A). The expression of *TNFSF11* positively correlated with the abundance of M1-subtype macrophages (R = 0.25; *p* = 0.028). The expression of *MITF* was significantly positively correlated with CD8^+^ T cells (R = 0.28; *p* = 0.012) and negatively correlated with resting NK cells (R = −0.24; *p* = 0.032). The expression of *CTNNB1* was positively correlated with memory B cells (R = 0.256; *p* = 0.022) and negatively correlated with naive B cells (R = −0.273; *p* = 0.014) (Figure 6B). 

We further analyzed human leukocyte antigen (HLA) gene sets extracted from the GSE56815 dataset; *HLA-DMB* and *HLA-DRB6* expression levels significantly differed between the high- and low-risk groups (Figure 6C). We found that *TNFSF11* expression was significantly negatively correlated with *HLA-DOB* expression (R = −0.04; *p* = 0.012); *MITF* expression negatively correlated with *HLA-DMB* (R = −0.005; *p* = 0.038) and positively correlated with *HLA-DOA* (R = 0.388; *p* = 0.028) expression; and *CTNNB1* expression positively correlated with *HLA-DOA* (R = 0.114; *p* = 0.023), *HLA-F* (R = 0.638; *p* = 0.037), and *HLA-G* (R = 0.752; *p* = 0.023) expression, and negatively correlated with *HLA-F-AS1* expression (R = −0.400; *p* = 0.020) (Figure 6D).

We next explored the differences in immune-response-related genes between the high- and low-risk groups and found that the expression levels of *LAG3*, *PDCD1*, and *CD244* differed significantly between the two groups (Figure 6E). *TNFSF11* expression was significantly positively correlated with *LAG3* (R = 0.230; *p* = 0.040), *CD244* (R = 0.228; *p* = 0.042), and *KLRG1* (R = 0.336; *p* = 0.002) expression. However, it was negatively correlated with *PDCD1* expression (R = −0.334; *p* = 0.003). *MITF* was significantly positively correlated with *CD27* (R = 0.237; *p* = 0.034), *PDCD1* (R = 0.274; *p* = 0.014), and *CTLA4* (R = 0.285; *p* = 0.011) expression, but negatively correlated with *CD244* (R = −0.427; *p* < 0.001). *CTNNB1* expression was negatively correlated with *CD7* expression (R = −0.290; *p* = 0.009) (Figure 6F).

### 3.6. Molecular Subtypes Mediated by Osteoclast-Regulatory Genes

**Figure 7:**
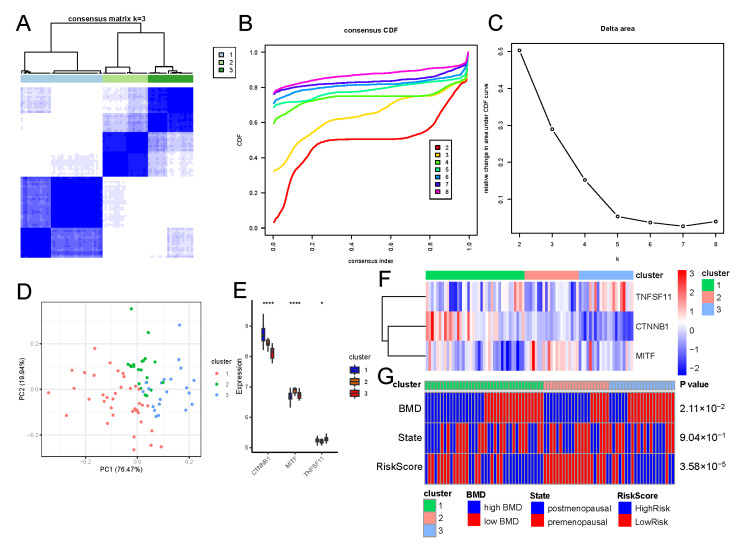
Molecular subtypes of osteoporosis mediated by osteoclast-regulatory genes. (**A**) Heatmap of sample clustering. (**B**) Cumulative distribution function curves for *CTNNB1*, *MITF*, and *TNFSF11*. (**C**) Delta area of *CTNNB1*, *MITF*, and *TNFSF11*. (**D**) Sample dimensionality reduction cluster plot. Peach: Cluster 1; green: Cluster 2; blue: Cluster 3. (**E**) Expression distribution of *CTNNB1*, *MITF*, and *TNFSF11* in the three molecular subtypes. The Kruskal–Wallis test was used to calculate the significance of differences in gene expression. Blue: Cluster 1; orange: Cluster 2; red: Cluster 3. (**F**) Heatmap of *CTNNB1*, *MITF*, and *TNFSF11* expression. (**G**) Distribution of clinicopathological features in molecular subtypes. * *p* < 0.05; **** *p* < 0.0001 (Chi-square test). Green: Cluster 1; pink: Cluster 2; light blue: Cluster 3; blue: high BMD/postmenopausal/high risk; red: low BMD/premenopausal/low risk. BMD: bone mineral density.

Based on the osteoclast-regulatory genes (*CTNNB1*, *MITF*, and *TNFSF11*) in the risk model, we used the R package “ConsensusClusterPlus” to cluster the osteoporosis patients (Figure 7A). Based on the cumulative distribution function and delta area plots, we found that K = 3 was a suitable value; therefore, we divided patients with osteoporosis into three subtypes (Figure 7B,C). Dimensionality reduction clustering showed that the patients in clusters 2 and 3 grouped into one class, with Cluster 1 mainly distributed at the bottom, demonstrating heterogeneity among subtypes (Figure 7D). Additionally, the expression patterns of osteoclast-regulatory genes in the three subtypes were characterized (Figure 7E,F). The risk gene *TNFSF11* was highly expressed in Cluster 3, whereas the protective genes *CTNNB1* and *MITF* were highly expressed in clusters 1 and 2, respectively. Finally, we characterized the clinical characteristics across molecular subtypes (Figure 7G). Cluster 3 had considerably more patients with osteoporosis with low bone density who were classified in the high-risk group, whereas Cluster 2 had considerably more patients with high bone density and those classified in the low-risk group, according to our model.

### 3.7. Immune Microenvironments of Different Molecular Subtypes

**Figure 8:**
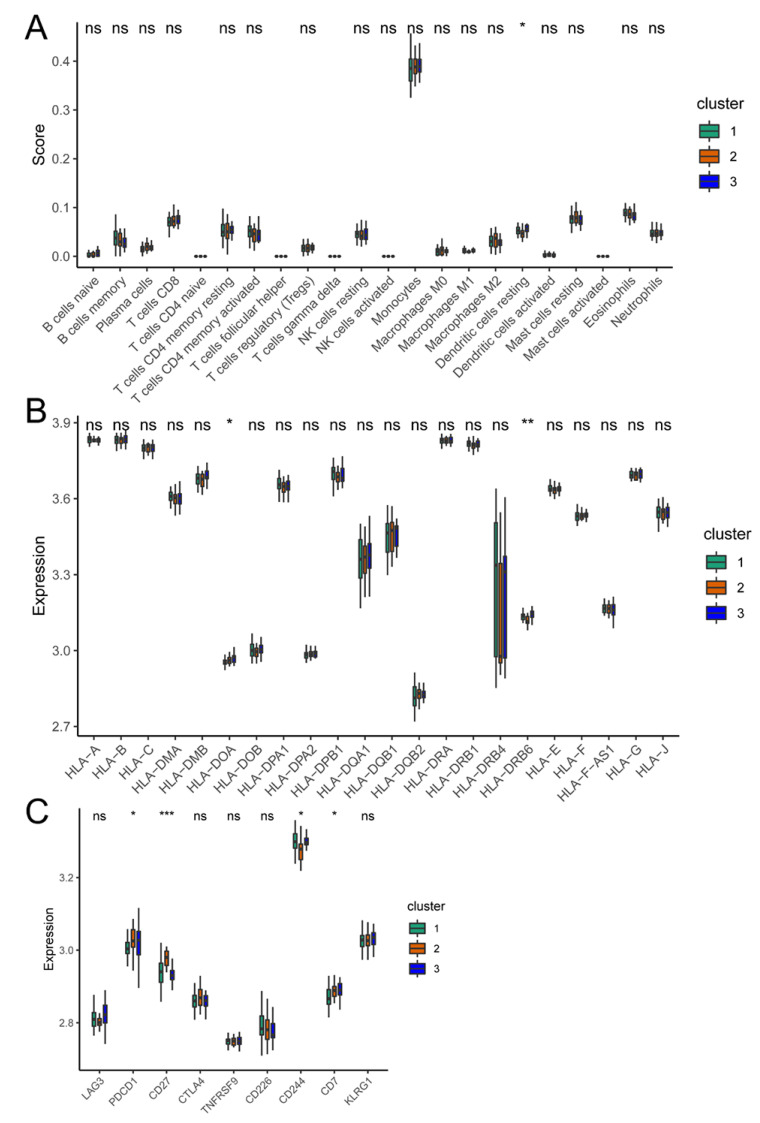
Immune microenvironments of different molecular subtypes of osteoporosis. (**A**) Differences in 22 immune cells among molecular subtypes of osteoporosis. (**B**) Expression differences in HLA-related genes among molecular subtypes. (**C**) Expression differences in immune-response-related genes among molecular subtypes. The Kruskal–Wallis test was used to calculate the significance of differences in gene expression. ns: no statistically significant difference; * *p* < 0.05; ** *p* < 0.01; *** *p* < 0.001. Green: Cluster 1; orange: Cluster 2; blue: Cluster 3.

To characterize the immune microenvironments of the three subtypes, the patterns of immune-infiltrating cells, immune response gene sets, and HLA-associated genes were investigated. The Cluster 3 subtype had considerably more dendritic cells (*p* < 0.05) (Figure 8A) and higher *HLA-DRB6* expression (*p* < 0.01) (Figure 8B) than the other clusters. *PDCD1* (*p* < 0.05), *CD27* (*p* < 0.001), *CD7* (*p* < 0.05), and other immune genes were highly expressed in Cluster 2, whereas *CD244* (*p* < 0.05) was highly expressed in Cluster 1 (Figure 8C). 

### 3.8. Functional Analysis of Different Molecular Subtypes

**Figure 9:**
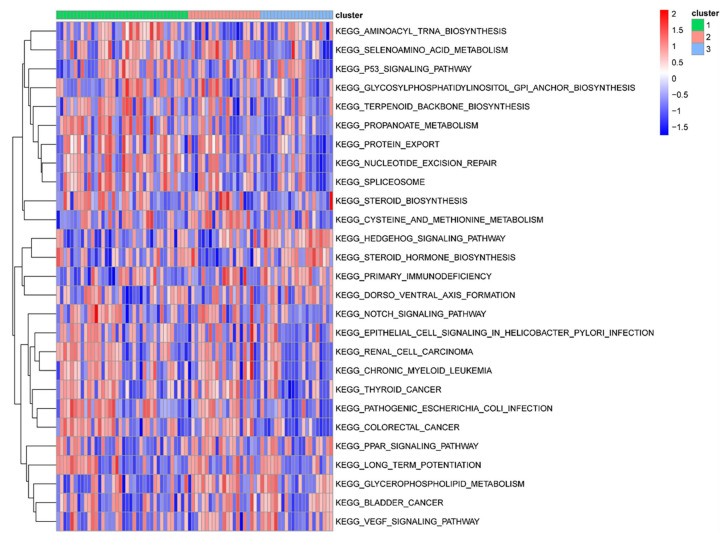
Functional analysis of different molecular subtypes. Heatmap of gene set variation analysis enrichment scores for different molecular subtypes. Green: Cluster 1; pink: Cluster 2; blue: Cluster 3.

The differential analysis of pathway enrichment scores identified 27 significantly different pathways between subtypes. We found that primary immunodeficiency and some metabolism-related pathways, including cysteine and methionine metabolism and glycerophospholipid metabolism, were mainly enriched in Cluster 2, indicating that this cluster has metabolism-related characteristics. Additionally, the hedgehog signaling and steroid hormone biosynthesis pathways were mainly enriched in Cluster 3 (Figure 9).

### 3.9. DEGs and Functional Analysis of Molecular Subtypes

**Figure 10:**
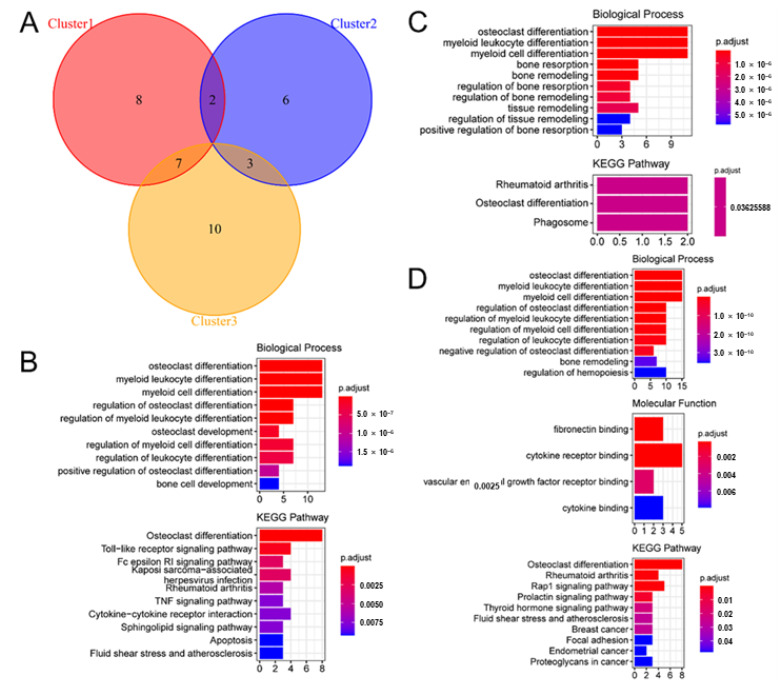
Differentially expressed genes and functional analysis of different molecular subtypes. (**A**) Venn diagram showing differentially expressed genes among different molecular subtypes of osteoporosis. Red: Cluster 1; blue: Cluster 2; yellow: Cluster 3. (**B**) Functions and pathways enriched by differentially expressed genes of Cluster 1. (**C**) Functions and pathways enriched by differentially expressed genes of Cluster 2. (**D**) Functions and pathways enriched by differentially expressed genes of Cluster 3.

Gene expression profiles were analyzed for differential expression between each subtype using the R package “limma”. A total of 17, 11, and 20 DEGs (*p* < 0.05) were identified in clusters 1, 2, and 3, respectively (Figure 10A). Functional enrichment analysis of these DEGs using the R package “clusterprofiler” showed that Cluster 1 was mainly enriched in osteoclast differentiation, myeloid cell differentiation, and Toll-like receptor signaling (Figure 10B); Cluster 2 was mainly enriched in osteoclast differentiation, myeloid cell differentiation, and phagosome maturation (Figure 10C); and Cluster 3 was mainly enriched in osteoclast differentiation, myeloid cell differentiation, and Rap1 signaling (Figure 10D). 

### 3.10. Potential Drug Targets Identified in the Protein–Protein Interaction (PPI) Network 

**Figure 11:**
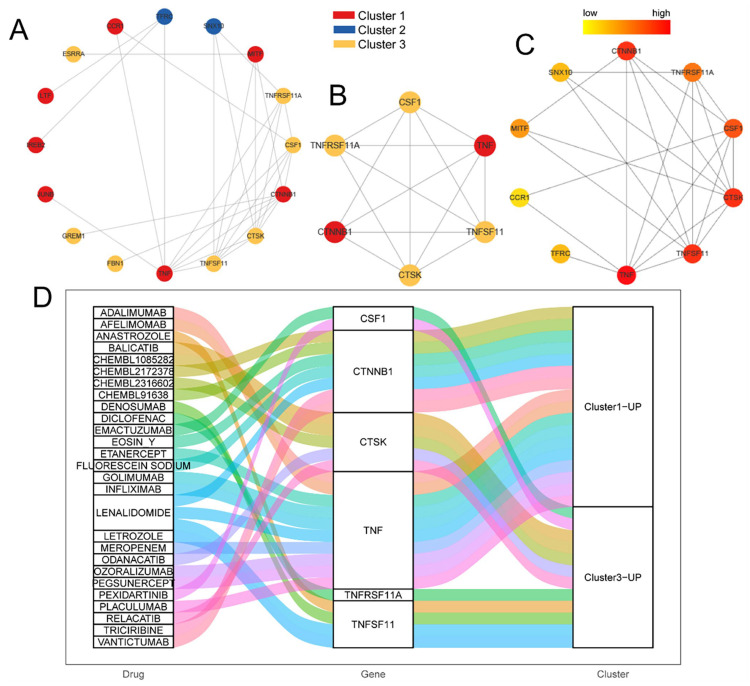
Potential drugs for osteoporosis based on hub nodes. (**A**) Protein–protein interaction network of upregulated genes for three molecular subtypes. (**B**) Key sub-networks of (**A**). (**C**) Degree of hub nodes in the network. (**D**) Sankey diagram showing drug–gene relationships. Red: Cluster 1; blue: Cluster 2; yellow: Cluster 3.

The upregulated genes in each subtype (25 upregulated genes in total) were mapped using the STRING database and the PPI network was reconstructed using Cytoscape (Figure 11A). *CTSK*, *CSF1*, *TNFSF11*, *CTNNB1*, *TNFRSF11A*, and *TNF* formed a key sub-network (Figure 11B), and were screened as key genes via cytoHubba according to the node degree of the network (Figure 11C). Therefore, these six genes were selected as the hub node, among which *TNF* and *CTNNB1* were the upregulated genes of Cluster 1, and *CTSK*, *CSF1*, *TNFSF11*, and *TNFRSF11A* were the upregulated genes of Cluster 3. The hub node was used as a query in DGIdb (https://dgidb.genome.wustl.edu/ accessed on 4 September 2023), which obtained 100 gene–drug interaction pairs, including 29 gene–drug interaction pairs with an interaction score >1 (Figure 11D). 

## 4. Discussion

In this study, we detected three key genes, *CTNNB1*, *MITF*, and *TNFSF11*, using microarray data for osteoporosis. These genes were selected as variables to construct a molecular risk model for predicting osteoporosis (risk score = Exp (*CTNNB1*) × (−1.167227) + Exp (*MITF*) × (−5.192496) + Exp (*TNFSF11*) × (12.019122)). Additionally, a molecular subtype classification map in postmenopausal women was drawn by analyzing these key genes. The results showed that Cluster 3 had significantly more patients with low BMD, whereas Cluster 2 had significantly more patients with high BMD. Furthermore, the immune microenvironment, DEGs, functional pathways, and gene–drug interactions of different molecular subtypes were described in detail. The strategy of our model complements the existing clinical assessment tools for screening high-risk postmenopausal women with osteoporosis, while the subtype classification also provides a novel perspective for further research.

Loss of mobility after a fracture is often a trigger for fatal events in older adults [22]. The high incidence and peculiar pre-fracture nature of osteoporosis emphasize the importance of screening for this disease. Dual-energy X-ray is a widely used and effective tool for diagnosing osteoporosis; however, it is not a practical screening tool. Although all women aged 65 years or older are recommended to undergo screening with DXA, nearly 25% of women aged 65–85 years never undergo a BMD test or have regular check-ups at the recommended frequency [10]. Additionally, studies have found that fewer DXA scans are performed in high-risk patients, whereas excessive DXA scans are performed in low-risk postmenopausal women [23]. A more detailed stratified strategy would be beneficial to complement age as a means of pre-screening for patients suitable for DXA.

Although rapid bone loss already occurs during the menopausal transition, there is no established risk assessment and screening strategy for younger postmenopausal women for osteoporosis [24]. The heterogeneity of biological genetic information among different populations may be an important factor in the progression of osteoporosis since some women develop osteoporosis early after menopause [25,26]. Risk assessment models of osteoporosis based on genetic information are rare, although several molecular models for other diseases have been developed [27,28,29]. This is partly because of the poor genetic information available on osteoporosis. Although some previous bioinformatics studies have also analyzed DEGs related to osteoporosis, in this study, we intended to further stratify the risk of osteoporosis in postmenopausal women posed by DEGs (Figure 4), and our findings revealed the differences in the immune infiltration microenvironment in patients with different molecular subtypes (Figure 8) [18,19]. Therefore, the molecular classification map obtained in this study can help to fill in these knowledge gaps and clinical needs. 

We identified three key genes using regression analysis (Figure 4). *CTNNB1* and *MITF* are protective factors, whereas *TNFSF11* is a risk factor for osteoporosis. *CTNNB1* is located at chromosome 3p22.1 and encodes important proteins for adherens junctions and the cytoskeleton system [30]. *MITF* is located at chromosome 3p13 and encodes a transcription factor that was first identified for its critical role in promoting the survival of migrating melanoblasts [31]. *TNFSF11* is located at 13q14.11 and encodes a member of the tumor necrosis factor cytokine family, RANKL, which is a ligand for the receptor activator of nuclear factor-κB and osteoprotegerin, directly related to osteoclast differentiation and activation [32,33]. 

Our molecular risk model complements existing clinical assessment strategies for osteoporosis; however, using this molecular model alone is not recommended. We believe that a risk model system that includes both genetic and environmental factors can be a better solution for a disease determined by both aspects such as osteoporosis [34]. Since genetic testing is an invasive operation, we need to first use a highly sensitive clinical assessment tool to screen perimenopausal women, and then, use this molecular model to comprehensively classify the risk of osteoporosis to determine the frequency of DXA examination for menopausal women. 

Owing to the limited knowledge of the pathophysiology of osteoporosis, cases are currently classified as “primary” or “secondary” based on largely unexplained clinical associations [35]. Our analysis revealed three molecular subtypes of primary osteoporosis according to differences in gene expression (Figure 7). Based on BMD levels in different groups, we speculate that the Cluster 3 subtype is more prone to a phenotype of low BMD with osteoporosis, Cluster 2 is a phenotype characterized by high BMD, and Cluster 1 showed an overall intermediate phenotype between clusters 2 and 3. Gene expression analysis supported this result; the risk gene *TNFSF11* was significantly highly expressed in Cluster 3 and the protective gene *MITF* was highly expressed in Cluster 2. We further characterized the functional pathways of the different subtypes, which may provide clues for further in-depth studies of primary osteoporosis.

Another focus of this study was on the immune infiltration microenvironment across different groups (Figure 8). Over the past two decades, important discoveries in the etiology of osteoporosis have demonstrated the deep integration of the skeletal system with the immune system, and the regulation of gene expression in immune cells is closely related to bone aging [36,37]. *PDCD1* is an important immunosuppressive molecule [38], and was found to be highly expressed in Cluster 2 and the low-risk group in this study. Greisen et al. [39] confirmed that *Pdcd1*-knockout mice showed signs of osteoporosis. Additionally, patients in Cluster 3 had significantly more dendritic cells, which are the most functional antigen-presenting cells [40]. These results provide insight into the role of the immune system in osteoporosis. Speculatively, autoimmunity may be an important factor in osteoporosis development. *TNFSF11*, which was upregulated in Cluster 3, is a dendritic cell survival factor involved in the regulation of T cell-dependent immune responses [41]. In contrast, T-cell activation was reported to induce the expression of *TNFSF11*, leading to an increase in osteoclast genesis and bone resorption [42].

We also analyzed the upregulated genes in each subtype and reconstructed the PPI network. *CTSK*, *CSF1*, *TNFSF11*, *CTNNB1*, *TNFRSF11A*, and *TNF* formed a key sub-network, and their corresponding gene–drug interactions were analyzed (Figure 11). *TNFRSF11A* is a target of diclofenac, an anti-inflammatory drug used to treat glaucoma, and *TNFSF11* is a target of lenalidomide, denosumab, and anastrozole. Denosumab has also been used to treat osteoporosis, whereas lenalidomide and anastrozole are antitumor drugs. *TNF* is a target of adalimumab and etanercept, both of which are antirheumatic drugs. *CTSK* is a target of drugs such as relacatib, which is an anti-osteoporosis drug that is not Food and Drug Administration-approved. *CSF1* is a target of pexidartinib, an antitumor agent. Therefore, these drugs may also be potential candidates for the treatment of osteoporosis. Furthermore, *CTSK*, *CSF1*, *TNFSF11*, *CTNNB1*, *TNFRSF11A*, and *TNF* may be the most promising osteoporosis markers.

This study has some limitations. First, although the analyzed public dataset represents the largest sample size for osteoporosis currently available, it is still insufficient to construct a more effective molecular risk model; thus, further studies with large sample sizes are needed as the cost of sequencing decreases. Second, since environmental factors also contribute to the development of osteoporosis, and gene expression is indeed partially driven by epigenetics, our molecular risk model only considers the genetic aspects of osteoporosis and is not recommended for stand-alone use. However, the design and validation of a risk assessment system that considers both genetic and environmental factors will require a considerably more comprehensive analysis that is beyond the scope of the present study. Third, we proposed three molecular subtypes and characterized their genes, functional pathways, and immune infiltration. However, further clarification of the differences and connections between the different subtypes needs to be provided by future experiments.

In conclusion, *CTNNB1*, *MITF*, and *TNFSF11* were identified as three key genes involved in the occurrence and development of osteoporosis. Our molecular risk model and subtype classification may complement existing clinical assessment tools and age-based strategies for pre-screening DXA-suitable patients. This evidence-based screening strategy will guide the active prevention, early diagnosis, and treatment of osteoporosis in high-risk postmenopausal women. Additionally, we identified the immune infiltration microenvironment in three molecular subtypes of osteoporosis, providing a novel perspective for further research.

## Figures and Tables

**Table 1:** Inclusion and exclusion criteria for participants in the external experimental validation study.

Inclusion Criteria	Exclusion Criteria
Confirmed diagnosis of osteoporosis according to WHO criteria (osteoporosis group)	Complications, including skeletal neoplasms, tuberculosis, infection, ankylosing spondylitis
Bone mineral density was determined as the lowest value in the lumbar spine and hip	Combined history of skeletal system surgery
Women ≥ 50 years old, <80 years old, and who were postmenopausal	Combined severe cardiopulmonary disease, severe liver or kidney dysfunction, untreated clotting disorders, and other major diseases
Primary osteoporosis (osteoporosis group)	Hypocalcemia or hypophosphatemia
Able to take care of themselves in daily life; Karnofsky performance status score ≥70	Combined connective tissue disease ^a^, endocrine and metabolic diseases ^b^, gastrointestinal and nutritional diseases, and hematological malignancy
Without previous anti-osteoporosis treatment	History of drug use affecting bone metabolism ^c^

^a^ Including systemic lupus erythematosus, rheumatoid arthritis, Sjogren’s syndrome, dermatomyositis, and mixed connective tissue disease. ^b^ Including hyperparathyroidism, Cushing’s syndrome, hypogonadism, hyperthyroidism, pituitary prolactinoma, type 1 diabetes mellitus, and hypopituitarism. ^c^ Including glucocorticoids, immunosuppressants, heparin, anticonvulsants, antineoplastic drugs, aluminum antacids, thyroid hormones, GnRH-a, or dialysate.

## Data Availability

We analyzed existing, publicly available data. The accession numbers for the datasets are listed in the key resources table. Any additional information required to reanalyze the data reported in this paper is available from the lead author upon request. No original code was used in this study.

## References

[1] Compston J.E., McClung M.R., Leslie W.D. (2019). Osteoporosis. Lancet.

[2] Kanis J.A. (1994). Assessment of fracture risk and its application to screening for postmenopausal osteoporosis: Synopsis of a WHO report. WHO study group. Osteoporos. Int..

[3] El Saman A., Meier S., Sander A., Kelm A., Marzi I., Laurer H. (2013). Reduced loosening rate and loss of correction following posterior stabilization with or without PMMA augmentation of pedicle screws in vertebral fractures in the elderly. Eur. J. Trauma Emerg. Surg..

[4] Wang L., Yu W., Yin X., Cui L., Tang S., Jiang N., Cui L., Zhao N., Lin Q., Chen L. (2021). Prevalence of osteoporosis and fracture in China: The China Osteoporosis Prevalence Study. JAMA Netw. Open.

[5] Kado D.M., Duong T., Stone K.L., Ensrud K.E., Nevitt M.C., Greendale G.A., Cummings S.R. (2003). Incident vertebral fractures and mortality in older women: A prospective study. Osteoporos. Int..

[6] Burge R., Dawson-Hughes B., Solomon D.H., Wong J.B., King A., Tosteson A. (2007). Incidence and economic burden of osteoporosis-related fractures in the United States, 2005–2025. J. Bone Miner. Res..

[7] Nayak S., Roberts M.S., Greenspan S.L. (2011). Cost-effectiveness of different screening strategies for osteoporosis in postmenopausal women. Ann. Intern. Med..

[8] Ruaro B., Casabella A., Paolino S., Alessandri E., Patané M., Gotelli E., Sulli A., Cutolo M. (2020). Trabecular bone score and bone quality in systemic lupus erythematosus patients. Front. Med..

[9] Trzaskowska M., Vivcharenko V., Kazimierczak P., Wolczyk A., Przekora A. (2023). In vitro screening studies on eight commercial essential oils-derived compounds to identify promising natural agents for the prevention of osteoporosis. Biomedicines.

[10] Ensrud K.E., Crandall C.J. (2017). Osteoporosis. Ann. Intern. Med..

[11] US Preventive Services Task Force (2011). Screening for osteoporosis: U.S. Preventive Services Task Force recommendation statement. Ann. Intern. Med..

[12] Rud B., Hilden J., Hyldstrup L., Hróbjartsson A. (2009). The Osteoporosis Self-Assessment Tool versus alternative tests for selecting postmenopausal women for bone mineral density assessment: A comparative systematic review of accuracy. Osteoporos. Int..

[13] Koh L.K., Sedrine W.B., Torralba T.P., Kung A., Fujiwara S., Chan S.P., Huang Q.R., Rajatanavin R., Tsai K.S., Park H.M. (2001). A simple tool to identify asian women at increased risk of osteoporosis. Osteoporos. Int..

[14] Cadarette S.M., Jaglal S.B., Kreiger N., McIsaac W.J., Darlington G.A., Tu J.V. (2000). Development and validation of the Osteoporosis Risk Assessment Instrument to facilitate selection of women for bone densitometry. CMAJ.

[15] Rubin K.H., Friis-Holmberg T., Hermann A.P., Abrahamsen B., Brixen K. (2013). Risk assessment tools to identify women with increased risk of osteoporotic fracture: Complexity or simplicity? A systematic review. J. Bone Miner. Res..

[16] Musumeci M., Vadalà G., Tringali G., Insirello E., Roccazzello A.M., Simpore J., Musumeci S. (2009). Genetic and environmental factors in human osteoporosis from Sub-Saharan to Mediterranean areas. J. Bone Miner. Metab..

[17] Boyle W.J., Simonet W.S., Lacey D.L. (2003). Osteoclast differentiation and activation. Nature.

[18] Hu M., Zou L., Lu J., Yang Z., Chen Y., Xu Y., Sun C. (2021). Construction of a 5-feature gene model by support vector machine for classifying osteoporosis samples. Bioengineered.

[19] Li J.J., Wang B.Q., Fei Q., Yang Y., Li D. (2016). Identification of candidate genes in osteoporosis by integrated microarray analysis. Bone Joint Res..

[20] Porcu E., Sadler M.C., Lepik K., Auwerx C., Wood A.R., Weihs A., Sleiman M.S.B., Ribeiro D.M., Bandinelli S., Tanaka T. (2021). Differentially expressed genes reflect disease-induced rather than disease-causing changes in the transcriptome. Nat. Commun..

[21] Fischer V., Haffner-Luntzer M. (2022). Interaction between bone and immune cells: Implications for postmenopausal osteoporosis. Semin. Cell Dev. Biol..

[22] Doostmohammadi A., Karimzadeh Esfahani Z., Ardeshirylajimi A., Rahmati Dehkordi Z. (2019). Zirconium modified calcium-silicate-based nanoceramics: An in vivo evaluation in a rabbit tibial defect model. Int. J. Appl. Ceram. Technol..

[23] Cunningham G.F. (2005). Screening for osteoporosis. N. Engl. J. Med..

[24] Lizneva D., Yuen T., Sun L., Kim S.M., Atabiekov I., Munshi L.B., Epstein S., New M., Zaidi M. (2018). Emerging concepts in the epidemiology, pathophysiology, and clinical care of osteoporosis across the menopausal transition. Matrix Biol..

[25] Lobo R.A., Gompel A. (2022). Management of menopause: A view towards prevention. Lancet Diabetes Endocrinol..

[26] Gosset A., Pouillès J.M., Trémollieres F. (2021). Menopausal hormone therapy for the management of osteoporosis. Best Pract. Res. Clin. Endocrinol. Metab..

[27] Hong H.C., Chuang C.H., Huang W.C., Weng S.L., Chen C.H., Chang K.H., Liao K.W., Huang H.D. (2020). A panel of eight microRNAs is a good predictive parameter for triple-negative breast cancer relapse. Theranostics.

[28] Long S.Y., Sun J.Y., Wang L., Long H., Jiang H.Q., Shi Y., Zhang W.Y., Xiong J.S., Sun P.W., Chen Y.Q. (2021). Predictive nomogram for leprosy using genetic and epidemiological risk factors in Southwestern China: Case-control and prospective analyses. EBiomedicine.

[29] Hachiya T., Kamatani Y., Takahashi A., Hata J., Furukawa R., Shiwa Y., Yamaji T., Hara M., Tanno K., Ohmomo H. (2017). Genetic predisposition to ischemic stroke: A polygenic risk score. Stroke.

[30] Yan D., Sun Y., Xu N., Yu Y., Zhan Y., Mainland Chinese League of NEDSDV Rare Disease (2022). Genetic and clinical characteristics of 24 mainland Chinese patients with CTNNB1 loss-of-function variants. Mol. Genet. Genom. Med..

[31] Chauhan J.S., Hölzel M., Lambert J.P., Buffa F.M., Goding C.R. (2022). The MITF regulatory network in melanoma. Pigment Cell Melanoma Res..

[32] Odgren P.R., Kim N., MacKay C.A., Mason-Savas A., Choi Y., Marks S.C. (2003). The role of RANKL (TRANCE/TNFSF11), a tumor necrosis factor family member, in skeletal development: Effects of gene knockout and transgenic rescue. Connect. Tissue Res..

[33] Gong Y., Bu Y., Li Y., Hao D., He B., Kong L., Huang W., Gao X., Zhang B., Qu Z. (2022). Hydrogel-based delivery system applied in the local anti-osteoporotic bone defects. Front. Bioeng. Biotechnol..

[34] Seddon J.M., Reynolds R., Yu Y., Rosner B. (2013). Validation of a prediction algorithm for progression to advanced macular degeneration subtypes. JAMA Ophthalmol..

[35] Mundy G.R. (2000). Secondary osteoporosis: The potential relevance of leptin and low body weight. Ann. Intern. Med..

[36] Weitzmann M.N., Ofotokun I. (2016). Physiological and pathophysiological bone turnover—Role of the immune system. Nat. Rev. Endocrinol..

[37] Li C.J., Xiao Y., Sun Y.C., He W.Z., Liu L., Huang M., He C., Huang M., Chen K.X., Hou J. (2021). Senescent immune cells release grancalcin to promote skeletal aging. Cell Metab..

[38] Wang Z., Wu X. (2020). Study and analysis of antitumor resistance mechanism of PD1/PD-L1 immune checkpoint blocker. Cancer Med..

[39] Greisen S.R., Kragstrup T.W., Thomsen J.S., Hørslev-Pedersen K., Hetland M.L., Stengaard-Pedersen K., Østergaard M., Ørnbjerg L., Junker P., Sharpe A.H. (2022). The programmed death-1 pathway counter-regulates inflammation-induced osteoclast activity in clinical and experimental settings. Front. Immunol..

[40] Zhou J., Li L., Jia M., Liao Q., Peng G., Luo G., Zhou Y. (2023). Dendritic cell vaccines improve the glioma microenvironment: Influence, challenges, and future directions. Cancer Med..

[41] Walsh M.C., Choi Y. (2021). Regulation of T cell-associated tissues and T cell activation by RANKL-RANK-OPG. J. Bone Miner. Metab..

[42] Bishop K.A., Wang X., Coy H.M., Meyer M.B., Gumperz J.E., Pike J.W. (2015). Transcriptional regulation of the human TNFSF11 gene in T cells via a cell type-selective set of distal enhancers. J. Cell. Biochem..

